# Twenty Years of Quantum State Teleportation at the Sapienza University in Rome

**DOI:** 10.3390/e21080768

**Published:** 2019-08-06

**Authors:** Francesco De Martini, Fabio Sciarrino

**Affiliations:** Dipartimento di Fisica—Sapienza Università di Roma, P.le Aldo Moro 5, I-00185 Roma, Italy

**Keywords:** quantum teleportation, entanglement, photonics, quantum information

## Abstract

Quantum teleportation is one of the most striking consequence of quantum mechanics and is defined as the transmission and reconstruction of an unknown quantum state over arbitrary distances. This concept was introduced for the first time in 1993 by Charles Bennett and coworkers, it has then been experimentally demonstrated by several groups under different conditions of distance, amount of particles and even with feed forward. After 20 years from its first realization, this contribution reviews the experimental implementations realized at the Quantum Optics Group of the University of Rome La Sapienza.

## 1. Introduction

Entanglement is today the core of many key discoveries ranging from quantum teleportation [1], to quantum dense coding [2], quantum computation [3,4,5] and quantum cryptography [6,7]. Quantum communication protocols such as device-independent quantum key distribution [8] are heavily based on entanglement to reach nonlocality-based communication security [9]. The prototype for quantum information transfer using entanglement as a communication channel is the quantum state teleportation (QST) protocol, introduced for the first time in 1993 by Charles Bennett et al. [1], where a sender and a receiver share a maximally entangled state which they can use to perfectly transfer an unknown quantum state. Undoubtedly, quantum teleportation is one of the most counterintituitive consequences of quantum mechanics and it is defined as the transmission and reconstruction over arbitrary distances of an unknown quantum state.

This protocol represents a milestone in theoretical quantum information science [10,11,12] and lies at the basis of many technological applications such as quantum communication via quantum repeaters [13,14] or gate teleportation [15]. Experimentally, this protocol has been demonstrated by several groups [16,17,18,19,20,21,22]. It has been implemented over hundreds of kilometers in free-space [23,24] and more recently in a ground-to-satellite experiment [25]. Employed platforms include mainly photonic qubits [16,17,18,20,21,22,26,27], but also nuclear magnetic resonance [28], atomic ensembles [29,30], trapped atoms [31,32] and solid-state systems [33,34,35]. The progress from the fundamental and technological point of view has continued, allowing the achievement of teleportation of multiple degrees of freedom of a single photon [27]. A quantum space race has started with the satellite-based distribution of entangled photon pairs to two locations, separated by 1203 kilometers on Earth [36], and the first satellite based quantum teleportation. Quantum teleportation experiments using deployed optical fibres for the distribution of entangled pairs have been also recently reported [37,38,39]: A key ingredient to build a quantum repeater. Quantum teleportation is a key primitive for quantum information processing, that has also been adopted for fundamental tests of quantum mechanics, such as one of the first loophole free Bell tests where entanglement swapping was exploited [40].

The present manuscript reviews the different experiments related to the teleportation of quantum states carried out in the Department of Physics, Sapienza University of Rome. In Section 2 we will briefly summarize the quantum teleportation protocol and its extension to entangled photon pairs: The so-called entanglement swapping. As the first experimental focus, in Section 3, we will describe the first quantum teleportation experiment performed in Rome. This scheme adopted two photons in order to encode the three qubits evolved in the QST protocol. This implementation had the unique capability to discriminate the four Bell states involved in an Alice node. We will then move to Section 4, where a different experimental approach has been adopted. There all the qubits are encoded in the vacuum-one photon Fock basis. This encoding further simplifies the required apparatus. The following step, described in Section 5, has then be to address a missing key ingredient in all the previous implementations of QST: The unitary operator $U_{i}$ to be implemented from Bob depending on the Alice outcome. This achievement was accomplished again, exploiting the concept of the vacuum-one photon qubit. This realization completed the contributions on the implementation of the original QST scheme. However, new scenarios arose from the modification of the teleportation. In 2004, we could identify how to modify the QST procedure in order to implement two fundamental optimal quantum machines: the universal NOT gate, and the universal optimal quantum cloning: these Universal Quantum Machines are discussed in Section 6. Finally, the last two sections are Section 7 and Section 8 are, respectively, devoted to a brief discussion on how to address the classical-quantum transition exploiting optimal quantum cloners, and on the future perspective of teleportation within quantum networks.

## 2. Teleportation and Entanglement Swapping

### 2.1. Quantum Teleportation Protocol

**Figure 1:**
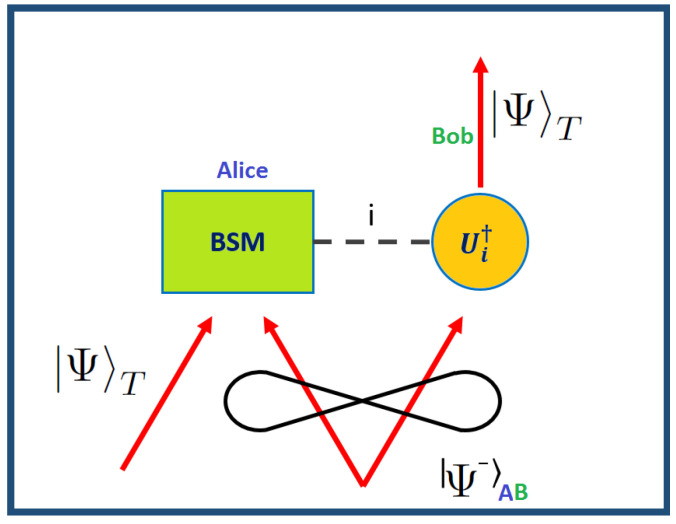
Pictorial representation of a teleportation protocol. Description of a teleportation protocol. The two stations A and B share an entangled state and a classical communication channel (dashed black line), which is used to communicate the result *i* of the Bell state measurement (BSM) performed in A in order to drive a unitary operation $U_{i}$. The initial quantum state ${|\Psi \rangle }_{T}$, which is physically present in A, is thus teleported in B.

The scheme of the teleportation protocol is depicted in Figure 1. By exploiting one maximally entangled state of the Bell basis, we can perform an experimental protocol of quantum teleportation, where two parties, named respectively Alice and Bob, are involved. The photon *A* of the entangled pair ${|\Psi^{-}\rangle }_{A,B}=\frac{1}{\sqrt[{}]{2}}\left({|01\rangle }_{AB}-{|10\rangle }_{AB}\right)$ is sent to Alice and the other photon *B* to Bob in order to share an entangled state. Let us consider that an unknown quantum state ${|\Psi \rangle }_{T}=\alpha {|0\rangle }_{T}+\beta {|1\rangle }_{T}$ was sent to Alice in order to be teleported, then the state of the whole system can be written as:

(1) $$\begin{array}{ccc} {|\Psi \rangle }_{T}⊗{|\Psi^{-}\rangle }_{A,B}= & - & \frac{1}{2}{|\Psi^{-}\rangle }_{T,A}\left(\alpha {|0\rangle }_{B}+\beta {|1\rangle }_{B}\right) \end{array}$$

(2) $$\begin{array}{cc} \;\;\;\;\;\;\;\;- & \frac{1}{2}{|\Psi^{+}\rangle }_{T,A}\left(\alpha {|0\rangle }_{B}-\beta {|1\rangle }_{B}\right) \end{array}$$

(3) $$\begin{array}{cc} \;\;\;\;\;\;\;\;+ & \frac{1}{2}{|\Phi^{-}\rangle }_{T,A}\left(\alpha {|1\rangle }_{B}+\beta {|0\rangle }_{B}\right) \end{array}$$

(4) $$\begin{array}{cc} \;\;\;\;\;\;\;\;+ & \frac{1}{2}{|\Phi^{+}\rangle }_{T,A}\left(\alpha {|1\rangle }_{B}-\beta {|0\rangle }_{B}\right). \end{array}$$

At this point in order to transfer the unknown state to Bob’s particle we must perform a Bell state measurement (BSM) in Alice’s station. The BSM is a projective measurement in the Bell basis, able to discriminate among the four two-mode entangled states. After the measurement performed by Alice, the state on Bob’s side is $U_{i}{|\Psi \rangle }_{T}$. The result of the measurement performed by Alice must be communicated to Bob by means of a classical channel and, according to this one, Bob applies the appropriate Pauli operations ($\sigma_{x}$, $\sigma_{z}$, $\sigma_{y}$ or nothing) on his station to complete the teleportation process and retrieve the input state since $U_{i}^{†}U_{i}{|\Psi \rangle }_{T}={|\Psi \rangle }_{T}$. Without the communication of the BSM result the particle *B* would be described by a fully mixed state.

### 2.2. Entanglement-Swapping Protocol

Is it possible to get entanglement between particles which have never interacted in the past? This simple question has motivated many theoretical and experimental works [1,41,42]. In order to better explain this protocol we will need four parties: Alice, Bob, Victor and Thomas. Let Alice share a maximally entangled state ${|\Phi^{+}\rangle }_{AB}=\frac{1}{\sqrt[{}]{2}}\left({|00\rangle }_{AB}+{|11\rangle }_{AB}\right)$ with Bob while Victor and Thomas share the same state between them ${|\Phi^{+}\rangle }_{VT}$. At this point the state of the whole system can be written as:

(5) $${|\Phi^{+}\rangle }_{AB}⊗{|\Phi^{+}\rangle }_{VT}.$$

The previous state can be designed in such a way that the particles of Alice and Thomas have never interacted before. If Bob and Victor perform a Bell state measurement (BSM), it turns out that for any of the outcomes the particles of Alice and Thomas will collapse to some Bell state. By exploiting classical communication Bob and Victor can send Thomas the measurement outcomes, and then Thomas can perform local rotations in order to obtain the entangled state ${|\phi^{+}\rangle }_{AT}$. After the BSM the particles of Alice and Thomas become entangled although they have never interacted directly before as they can be created by different sources in highly separated places. One sees that this protocol is basically an extension of the teleportation one, where one member of the first Einstein–Podolsky–Rosen (EPR) pair (between Alice and Bob) is teleported to the second EPR pair (between Victor and Thomas). We must keep in mind that any pair can be chosen as the teleported pair or the channel. The idea of entanglement swapping was developed in order to distribute the entanglement over long distances, this is a fundamental feature to implement a quantum repeater [43]. This idea was also generalized to multipartite scenarios [44] which are particularly useful when working with quantum cryptography.

## 3. The First Teleportation

Following the original teleportation paper and its continuous-variables version, an intensive experimental effort started for the experimental realization of teleportation. Here we focus on the first experiment carried out in Rome. As recently reported by Nicolas Gisin in Nature [45]:

*"Two groups achieved the feat of quantum teleportation in 1997—just four years after the theoretical breakthrough. First, it was the team of Boschi et al. based in Italy, followed only a few months later by the team of Bouwmeester et al. in Austria."*

**Figure 2:**
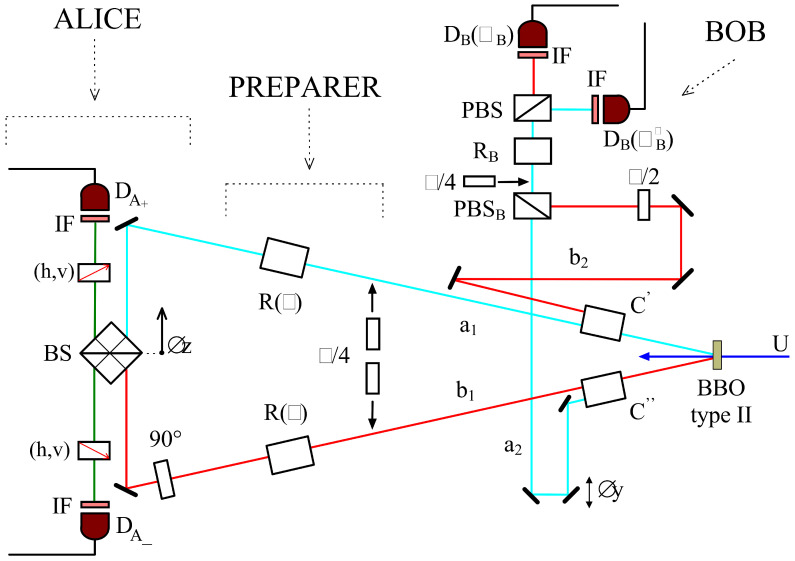
Experimental scheme adopted in the 1998 experiment showing the separate roles of the preparer, Alice and Bob. Pairs of polarization entangled photons are created directly using type II degenerate parametric down-conversion. By means of quarter wave plates acting in the same way on paths $a_{1}$ and $b_{1}$ the polarization degree of freedom of photon 1 is used to prepare the state to be teleported. For Alice, the polarization of path $b_{1}$ is first rotated by a further $90^{\circ }$. Then paths $a_{1}$ and $b_{1}$ impinge on the two input ports of an ordinary 50:50 beamsplitter (BS). At this beamsplitter each of the two polarizations *h*, and *v* interfere independently. After the beamsplitter there are two polarizers which are set either to transmit *h* or to transmit *v* polarization to the detector $D_{A_{\pm }}$. At Bob’s end, path $b_{2}$ is rotated through $90^{\circ }$ by a half waveplate. The paths $a_{2}$ and $b_{2}$ are combined at a polarizing beamsplitter orientated to transmit vertical and reflect horizontal polarization, then letting it impinge on a polarizing beamsplitter followed by two detectors $D_{B}\left(\theta_{B}\right)$. PBS, IF, and BBO stand, respectively, for Polarizing Beam Splitter, Interferential Filter and Beta Barium Borate. Picture from [17].

The scheme adopted in Rome exploited the approach proposed by Sandu Popescu in 1995 [46]. A total of two photons, rather than three as done in Innsbruck by Zeilinger’s group [16], were used. Let us briefly summarize the description provided in Figure 2. The two photon entangled state exhibited a path entanglement while the polarization degree of freedom of one of the photons was employed for preparing the unknown state. This approach avoided the difficulties associated with having three photons, as done in [16], and made the Bell measurement complete. This scheme is equivalent to the original scheme up to a local operation (since, in principle, any unknown state of a particle from outside could be swapped onto the polarization degree of freedom of Alice’s EPR particle by a local unitary operation as discussed below). In particular, if the preparer does not tell Alice what state he has prepared then there is no way Alice can find out what the state is. It is worthwhile mentioning that this approach leads to a 100% success rate for the Bell measurement in the ideal case rather than 50% as in three photon based schemes.

The scheme adopted in the experimental realization is reported in Figure 2. Pairs of polarization entangled photons were created directly using type-II degenerate parametric down-conversion. The article reported results for the teleportation of a linearly polarized state and of an elliptically polarized state. It showed that the experimental results cannot be explained in terms of a classical channel alone. The Bell measurement could distinguish between all four Bell states simultaneously allowing, in the ideal case, a 100% success rate of teleportation. As said, this scheme exploited two degrees of freedom of the same particle (polarization and path) to implement two different qubits: This approach allows the achievement of a deterministic Control-NOT gate leading to a complete Bell state measurement apparatus.

Let us note that the merging of the polarization quantum state of two photons into one photon has been recently reported by the Roma group in collaboration with the University of Naples Federico II [47]. This physical process has been named ‘quantum joining’, in which the two-dimensional quantum states of two input photons are combined into a single output photon, within a four-dimensional Hilbert space. This process provides a flexible quantum interconnect to bridge multi-particle protocols of quantum information with multidegree-of-freedom ones. Hence, by exploiting the quantum joining, it is possible to join the quantum state to be teleported with the photon *A* of the entangled pair. By this approach it is then possible to teleport any external quantum states via the “Roma” teleportation scheme. The scheme demonstrated in [47] is probabilistic with a success probability equal to 1/8, to be compared with the success probability of 1/2 for the scheme adopted by [16]. Nevertheless it is possible to enhance the merging probability up to 1 by increasing the number of ancillary photons and then the complexity of the related scheme [48]. Alternatively, by adopting gigantic nonlinear interactions among photons currently under development [49], deterministic schemes for quantum-state joining and splitting should also become possible [48].

The experiment carried out in Rome was submitted to Physical Review Letters on 28 July 1997 and posted on arXiv 2 October 1997 [17]. We refer to [50] for a complete comparison between the experiments carried out in Rome [17] and Innsbruck [16]. Here we are not describing how to adapt the QST protocol to continuous variable systems [26]: a very exhaustive review on these concept and implementations can be found in [12]. We refer to [51] and [12] for an exhaustive description of Quantum Teleportation with Continuous Variables. It is worth mentioning that a long debate has addressed the differences between unconditional (or deterministic) and conditional quantum teleportation: the different points of view, respectively, of the continuous and discrete variable community are properly summarized in [12] and [52].

## 4. Teleportation of Vacuum-One Photon

The Roma team addressed a qubit teleportation with a large fidelity by adopting the concept of entanglement of one photon with the vacuum [18]. The underlying motivation was to identify and implement the simplest scheme to observe the essence of the teleportation of a quantum state. By this approach, each quantum superposition state, i.e., a qubit, was physically implemented by a two dimensional subspace of Fock states of a mode of the electromagnetic field, specifically the space spanned by the “vacuum” and the 1-photon state. In other words, the field’s modes rather than the photons associated with them have been properly taken as the information and entanglement carriers.

The following details are taken from reference [53], where a complete description of the scheme and related experiment is available. If A and B represent two different modes of the field, with wavevectors $k_{A}$ and $k_{B}$ directed respectively towards two distant stations (Alice and Bob), these ones may be linked by a non-local channel expressed by an entangled state implying the quantum superposition of a single photon, e.g., by the singlet: ${|\Psi^{-}\rangle }_{A,B}=\frac{1}{\sqrt[{}]{2}}\left({|0\rangle }_{kA}{|1\rangle }_{kB}-{|1\rangle }_{kA}{|o\rangle }_{kB}\right)$ Here the mode indexes 0 and 1 denote respectively the vacuum and 1-photon Fock state population of the modes $k_{A}$, $k_{B}$ and the superposition state may be simply provided by an optical beam splitter (BS), as we shall see.

Conceptually this experiment represents one of the first (if not the first) application of “single-photon nonlocality”, a paradigm first introduced by Albert Einstein in a context close to the formulation of the Einstein-Podolsky-Rosen paradox [54] and later elaborated by [55,56]. Moreover this scheme is highly connected with single particle entanglement adopted as a key resource in the method proposed by Knill, Laflamme and Milburn [57] to implement universal quantum computing with linear optics.

Of course, in order to make use of the entanglement present in this picture we need to use the second quantization procedure of creation and annihilation of particles and/or use states which are superpositions of states with different numbers of particles. Another puzzling aspect of this second quantized picture is the need to define and measure the relative phase between states with different number of photons, such as the relative phase between the vacuum and one photon state. In order to control these relative phases we need, in analogy with classical computers, to supply all gates and all sender/receiving stations of a quantum information network with a common clock signal, e.g., provided by an ancillary photon or by a multi-photon, Fourier transformed coherent pulse.

The quantum system whose state we want to teleport is a qubit defined on the Hilbert space spanned by the vacuum state ${|0\rangle }_{S}$ and the one Fock-state ${|1\rangle }_{S}$ of the mode $k_{S}$. Thus the mode $k_{S}$ can be considered the qubit to be teleported. Suppose now that the qubit $k_{S}$ is in an arbitrary pure state $\alpha {|0\rangle }_{S}+\beta {|1\rangle }_{S}$. The overall state of the system and the non-local channel is then:

(6) Φtotal=2−12α0S+β1S1A0B−0A1B=2−12αΨ1SA1B+2−12αΨ2SA0B+=2−1Ψ3SAα0B+β1B+=2−1Ψ4SAα0B−β1B

where the states $|\Psi_{SA}^{j}\rangle$, j=(1,2,3,4) are defined below in Equations (7)–(10). The teleportation proceeds with Alice performing a partial Bell measurement. She combines the modes $k_{S}$ and $k_{A}$ on a symmetric beam splitter $BS_{A}$ whose output modes $k_{1}$ and $k_{2}$ are coupled to two detectors $D_{1}$ and $D_{2}$, respectively. As a consequence, we obtain

(7) $$\begin{array}{ccc} \;|\Psi_{SA}^{1}\rangle & = & {|0\rangle }_{S}{|0\rangle }_{A}={|0\rangle }_{1}{|0\rangle }_{2} \end{array}$$

(8) $$\begin{array}{ccc} \;\;\;\;\;\;\;|\Psi_{SA}^{2}\rangle & = & {|1\rangle }_{S}{|1\rangle }_{A}=2^{-\frac{1}{2}}\left({|2\rangle }_{1}{|0\rangle }_{2}-{|0\rangle }_{1}{|2\rangle }_{2}\right) \end{array}$$

(9) $$\begin{array}{ccc} \;\;\;\;\;\;\;|\Psi_{SA}^{3}\rangle & = & 2^{-\frac{1}{2}}\left({|0\rangle }_{S}{|1\rangle }_{A}-{|1\rangle }_{S}{|0\rangle }_{A}\right)={|1\rangle }_{1}{|0\rangle }_{2} \end{array}$$

(10) $$\begin{array}{ccc} \;\;\;\;\;\;\;|\Psi_{SA}^{4}\rangle & = & 2^{-\frac{1}{2}}\left({|0\rangle }_{S}{|1\rangle }_{A}+{|1\rangle }_{S}{|0\rangle }_{A}\right)={|0\rangle }_{1}{|1\rangle }_{2}. \end{array}$$

**Figure 3:**
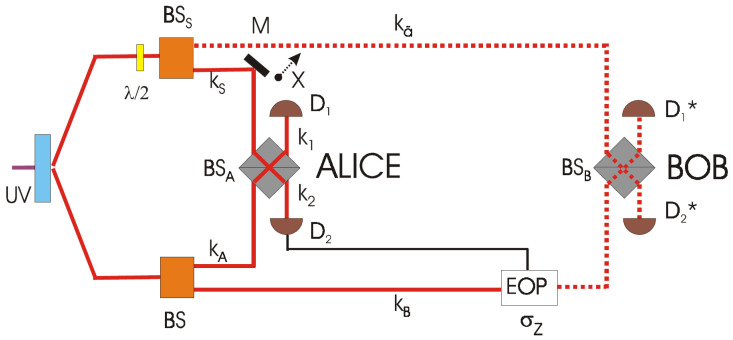
Experimental scheme adopted in the 2002 teleportation of a vacuum–one-photon qubit. EOP denotes a high-voltage Electro-Optic Pockels cell, BS denote beam splitter and D detectors. Picture from [18].

The state $|\Psi_{SA}^{3}\rangle$ is a Bell type state. From Equation (9) we see that its realization implies a single photon arriving at the detector $D_{1}$ and no photons at $D_{2}$ in Figure 3. Similarly, $|\Psi_{SA}^{4}\rangle$ is a Bell type state and it implies a single photon arriving at the detector $D_{2}$ and no photons at $D_{1}$. In both these cases the teleportation is successful. On the other hand, when Alice finds $|\Psi_{SA}^{1}\rangle$ or $|\Psi_{SA}^{2}\rangle$ the teleportation fails. From Equation (6) we see that teleportation is successful in 50% of the cases. By using appropriate entangled resources the teleportation step can be made near deterministic by means of linear optics, photon counting and fast feedforward.

## 5. Active Teleportation

Up to 2002 all the implementation of quantum state teleportation, including the one reported in the previous Sections, corresponded to simplified “passive” schemes where the transformation $U_{i}$ at Bob’s side was not implemented. In all these experiments the success of the protocol was demonstrated indirectly by the detection of the correlations established a posteriori between the extreme stations, Alice and Bob. These passive realizations had the advantage of avoiding the difficult implementation of the final stage of the protocol, i.e., of the unitary transformations $U_{i}$ restoring the exact input qubit at Bob’s site depending on the outcome of Alice’s Bell measurement. The main problem faced here was due to the relatively long time needed to activate, under single-photon excitation by Alice’s Bell-measurement apparatus, an Electro-Optic Pockels cell, which implements the necessary U-unitaries at Bob’s site. The following details are again taken from [53], where a complete description is available.

**Figure 4:**
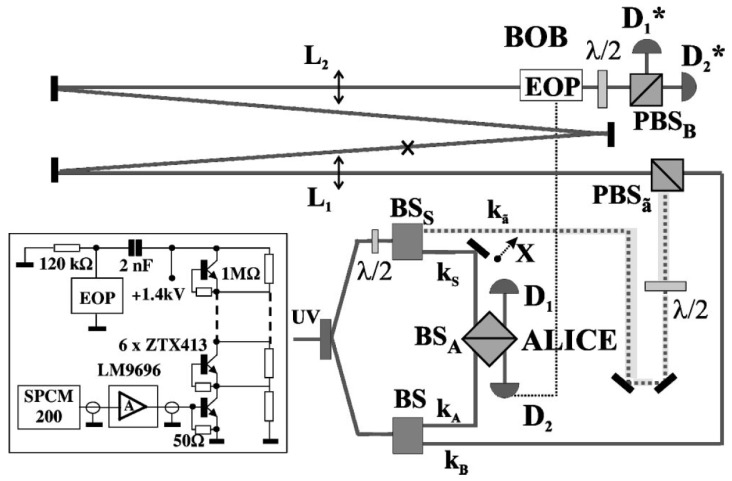
”Active” teleportation of a quantum bit. The experimental set-up can be somewhat considered to be the ”folded” configuration of the one reported in Figure 2. The significant changes consisted of the addition of the optical delay line (DL) and of a different measurement apparatus at Bob’s site where a high-voltage micro Electro Optics Pockels cell (EOP) performs a unitary transformation $U\equiv \sigma_{z}$. In the inset is reported the diagram of the fast electronic switch of (EOP). Picture from [19].

The work realized in [19] reported for the first time the complete, i.e., active, qubit teleportation process by completing the corresponding optical scheme according to the full quantum teleportation protocol. This achievement was accomplished exploiting the concept of vacuum-one photon qubit introduced in the previous section. The experimental setup, Figure 4, can be somewhat considered to be the “folded” configuration of the one reported in Figure 3. The significant changes consisted of the addition of the optical delay line and of a different measurement apparatus at Bob’s site.

## 6. Optimal Quantum Machines Based on Teleportation

In 2002, the focus of the Rome research activities moved from the implementation of quantum state teleportation protocol to the physical realization of different optimal quantum machines. Let us first briefly summarize the scientific background. We will then highlight the connection with the QST.

At a fundamental level quantum information (QI) consists of the set of rules that identify and characterize the physical transformations that are applicable to the quantum state of any information system. Because of the constraints established by the quantum rules it is found that several classical information tasks are forbidden or cannot be perfectly extended to the quantum world. A well known and relevant QI limitation consists of the impossibility of perfectly cloning (copying) any unknown qubit $|\phi \rangle$ [58]. In other words, the map $|\phi \rangle \to |\phi \rangle |\phi \rangle$ cannot be realized by nature because it does not belong to the set of completely positive (CP) maps. Another forbidden operation is the NOT gate that maps any $|\phi \rangle$ in its orthogonal state $|\phi^{⊥}\rangle$ [59]. Even if these two processes are unrealizable in their exact forms, they can be optimally approximated by the so-called universal optimal quantum machines, which exhibit the minimum possible noise.

**Figure 5:**
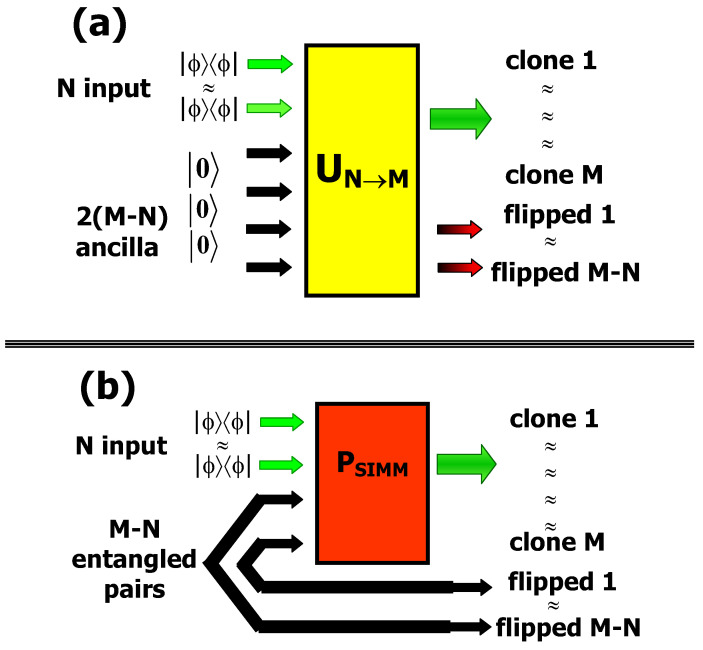
General scheme for the simultaneous realization of the universal NOT (UNOT) gate and of the universal quantum cloning machine. (**a**) Unitary transformation acting on the N-input qubits and 2(M-N) ancilla qubits initially in the state $|0\rangle$. (**b**) Symmetrization process acting on the input qubits and (M-N) entangled pairs of qubits. Picture partially from [68].

A complete understanding of these processes is important since the exact characterization of the quantum constraints within basically simple QI processes is useful to design more sophisticated algorithms and protocols and to assess the limit performance of complex networks. The efficiency of a gate, that measures how close its action is to the desired one, is generally quantified by the fidelity F.F=1 implies a perfect implementation, while noisy processes correspond to: F<1. The universal NOT (UNOT) gate, the optimal approximation of the NOT gate, maps N identical input qubits $|\phi \rangle$ into *M* optimal flipped ones in the state $\sigma_{out}$. It achieves the fidelity: $F_{N\to M}^{*}\left(|\phi^{⊥}\rangle ,\sigma_{out}\right)=\langle \phi^{⊥}|\sigma_{out}|\phi^{⊥}\rangle =\left(N+1\right)/\left(N+2\right)$ that depends only on the number of the input qubits [60]. Indeed the fidelity of the UNOT gate is exactly the same as the optimal quantum estimation fidelity [61]. This means that such process may be modeled as a “classical”, i.e., exact, preparation of *M* identical flipped qubits following the quantum, i.e., inexact, estimation of *N* input states. Only this last operation is affected by noise. Differently from the UNOT gate, the universal optimal quantum cloning machine (UOQCM), which transforms *N* identical qubits $|\phi \rangle$ into *M* identical copies $\rho_{out}$, achieves as optimal fidelity: $F_{N\to M}\left(|\phi \rangle ,\rho_{out}\right)=\langle \phi |\rho_{out}|\phi \rangle =\left(N+1+\beta \right)/\left(N+2\right)$ with β=N/M≤1 [62,63,64]. As we can see $F_{N\to M}\left(|0\rangle ,\rho_{out}\right)$ is larger than the one obtained by the *N* estimation approach and reduces to that result for β→0, i.e., for an infinite number of copies. The extra positive term β in the above expression accounts for the excess of quantum information which is originally stored in *N* states and is optimally redistributed by entanglement among the M−N remaining blank qubits encoded by UOQCM. The UNOT gate and the UOQCM can be implemented following two different approaches:

(i) The first one has been based on finding a suitable unitary operator $U_{NM}$, acting on *N* input qubits and on 2(M−N) ancillary qubits: Figure 5a. At the output of this device we obtain M and M−N qubits which are, respectively, the optimal clones and the best flipped qubits of the input ones. The transformation $U_{NM}$ can be deterministically realized by means of a quantum network, as proposed by Buzek et al. [65].
(ii) The second approach to implement the N→M cloning and the N→(M−N) flipping is a probabilistic method that exploits a symmetrization process: Figure 5b. The initial state of the overall system consists of the N input qubits and of (M−N) pairs of entangled qubits. The two optimal quantum machines are performed by applying a projective operation on the symmetric subspace to the *N* input qubits and to (M−N) ancilla qubits, each one belonging to a different entangled pair. This scheme corresponds to a modified QST scheme: Instead of performing a Bell state measurement a project over the symmetric subspace is performed. This transformation assures the uniform distribution of the initial information into the overall system and guarantees that all output clone qubits are indistinguishable. The success probability is equal to $\frac{1}{2^{M-N}}\frac{1+M}{1+N}$. The (M−N) optimal flipped qubits are teleported in a different location since there is no interaction between the N input qubits and the (M−N) flipped ones.

### Qubits Symmetrization: Linear Optics Implementation

Let us consider the scenario where there is N=1 initial qubit and the goal is to obtain M=2 optimal clones and 1 optimal flipped qubit.

The protocol that realizes the 1→2 UOQCM and 1→1 Tele-UNOT gate, involves two distant partners: Alice (A) and Bob (B). A holds the unknown input qubit S in a generic state ${|\phi \rangle }_{S}$, while B shall finally receive this qubit encoded optimally by the UNOT transformation of ${|\phi \rangle }_{S}$. Let A and B share the entangled singlet state of two qubits A,B: ${|\Psi^{-}\rangle }_{AB}=2^{1/2}\left({|\phi \rangle }_{A}{|\phi^{⊥}\rangle }_{B}-{|\phi^{⊥}\rangle }_{A}{|\phi \rangle }_{B}\right)$, as in a quantum teleportation protocol [1]. The choice of the singlet state guarantees, in virtue of its SU(2) invariance, the universality of the overall process. The overall state of the system reads ${|\Omega \rangle }_{SAB}=2^{-1/2}{|\phi \rangle }_{S}\left({|\phi \rangle }_{A}{|\phi^{⊥}\rangle }_{B}-{|\phi^{⊥}\rangle }_{A}{|\phi \rangle }_{B}\right)$. Let A to apply to the overall initial state ${|\Omega \rangle }_{SAB}$ the projective operator $P_{SA}$ over the symmetric subspace of the qubits S and A:

(11) $$P_{SA}=(I_{SA}-{|\Psi^{-}\rangle }_{SA}\langle \Psi^{-}{|}_{SA}).$$

The projection is successfully realized with probability p=3/4. In this case the normalized output state is:

(12) $$\begin{array}{c} {|\Theta \rangle }_{SAB}=\sqrt[{}]{2/3}{|\phi \rangle }_{S}{|\phi \rangle }_{A}{|\phi^{⊥}\rangle }_{B}\\ -\frac{1}{\sqrt[{}]{6}}\left({|\phi \rangle }_{S}{|\phi^{⊥}\rangle }_{A}+{|\phi^{⊥}\rangle }_{S}{|\phi \rangle }_{A}\right){|\phi \rangle }_{B}. \end{array}$$

One bit of classical communication sent by A announces to B the success of the symmetrization protocol. Note that the presence of the entangled state ${|\Psi^{-}\rangle }_{AB}$ is not strictly necessary for the sole implementation of the quantum cloning process. Indeed, for this purpose, we could apply $P_{SA}$ to the initial state ${|\phi \rangle }_{S}{\langle \phi |}_{S}⊗\frac{I_{A}}{2}$ as shown in [66,67].

**Figure 6:**
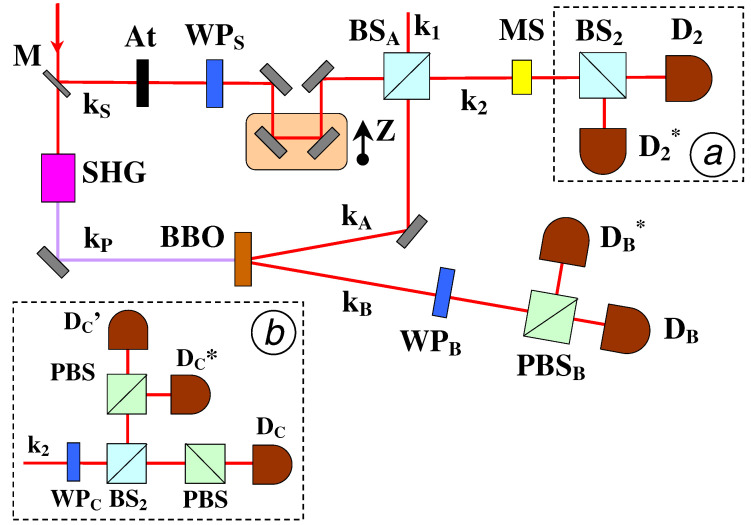
Setup for the optical implementation of the Tele-UNOT Gate and the probabilist universal optimal quantum cloning machine (UOQCM). The measurement setups used for the verification of the cloning and Universal NOT gate experiments are reported, respectively, in the inset (**b**) and (**a**). At, WP, MS, SHG, M, PBS denote, respectively, Attenuation Filter, Wave Plate, Mode Selector, Second Harmonic Generation, Partially Reflecting Mirror, Polarizing Beam Splitter. Picture from [68].

In the experiments reported in [68] and [67], the input qubit was codified into the polarization state of a single photon belonging to the input mode $k_{S}:{|\phi \rangle }_{S}=\alpha {|H\rangle }_{S}+\beta {|V\rangle }_{S}$, whereas an entangled pair ${|\Psi^{-}\rangle }_{AB}$ of photons A and B, was generated on the modes $k_{A}$ and $k_{B}$ by spontaneous parametric down conversion (SPDC). The projective operation in the space $H=H_{A}⊗H_{S}$ was realized exploiting the linear superposition of the modes $k_{S}$ and $k_{A}$ generated by a 50:50 beam-splitter, $BS_{A}$ (Figure 6). This superposition allows a partial Bell measurement on the $BS_{A}$ output states which is needed to implement the cloning machine and the Tele-UNOT gate. Consider the overall output state realized on the two modes $k_{1}$ and $k_{2}$ of the $BS_{A}$ and expressed by a superposition of the Bell states: $\left({|\Psi^{-}\rangle }_{SA},{|\Psi^{+}\rangle }_{SA},{|\Phi^{-}\rangle }_{SA},{|\Phi^{-}\rangle }_{SA}\right)$. The realization of the singlet ${|\Psi^{-}\rangle }_{SA}$ is identified by the emission of one photon on each output mode of $BS_{A}$, while the realization of the other three Bell states implies the emission of two photons either on mode $k_{1}$ or on mode $k_{2}$. This Hong-Ou-Mandel interference process, expressing a Bose mode coalescence (BMC) of the two photons over the same mode, was experimentally identified by a coincidence event between two detectors coupled to the output mode $k_{2}$ by means of an additional 50:50 beam-splitter by a post-selection technique. The projection into the symmetric space lies at the core of the cloning process.

By this approach two relevant quantum information processes, forbidden by quantum mechanics in their exact form, have been found to be connected contextually by a modified quantum state teleportation scheme in an optimal way. The complete implementation of this protocol has been successfully performed by a fully linear optical setup, which has also been shown to be scalable to a larger number of particles.

## 7. Micro and Macro Entanglement

As a following step, the goal has been to extend the previous results to a larger number of particles. To this scope non-linear optics interaction have been exploited for an extended research focused on the theoretical and experimental realization of a macroscopic quantum superposition (MQS) made up of photons. This intriguing, fundamental quantum condition is at the core of the famous argument conceived by Schrodinger in 1935. One of the main experimental challenges to the actual realization of this object resides in unavoidable interactions with the environment, leading to the cancellation of any evidence of the quantum features associated with the macroscopic system.

**Figure 7:**
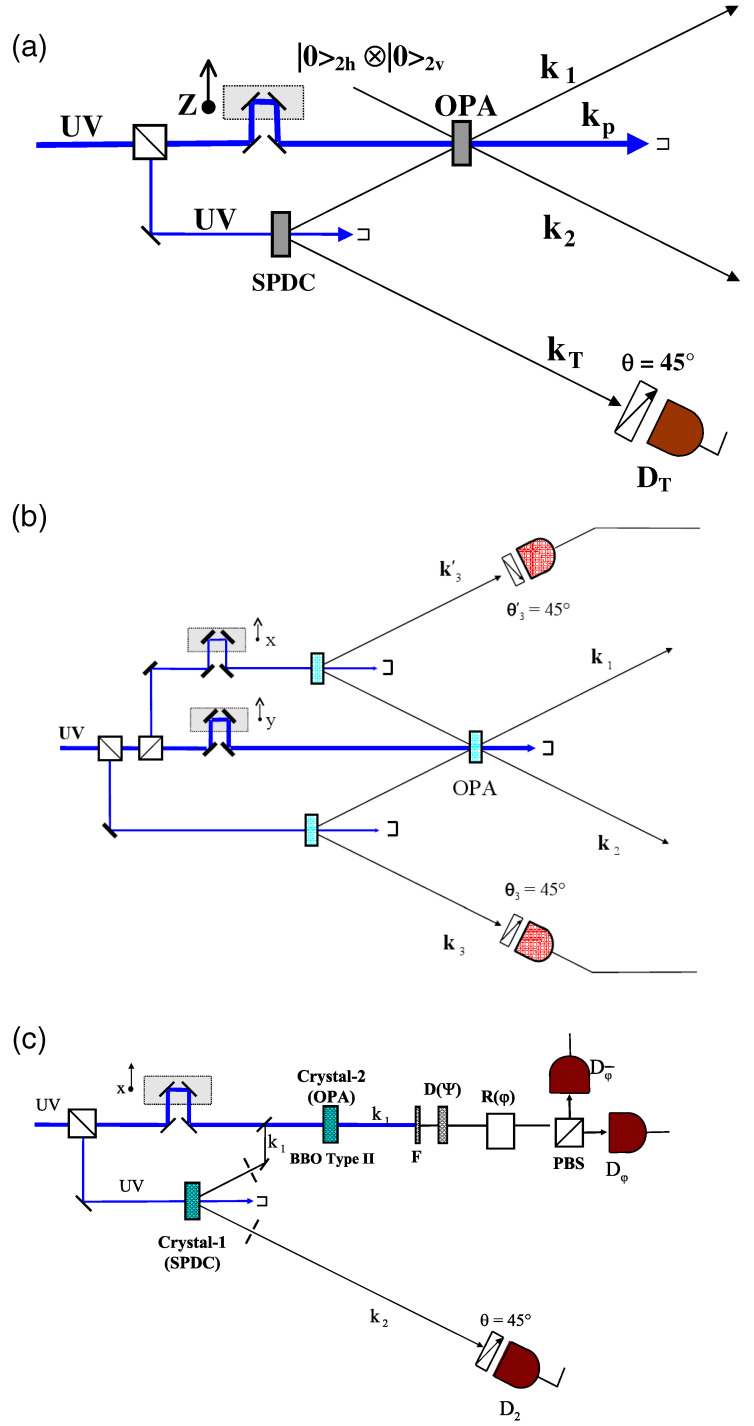
Three different configurations for the amplification of quantum states. (**a**) Schematic diagram of a noncollinear quantum-injected optical parametric amplifier (OPA). The injection is provided by an external spontaneous parametric downconversion source of polarization-entangled photon states. (**b**) Double injection of the optical parametric amplifier. (**c**) Collinear quantum-injected optical parametric amplifier. Picture from [69]. Spontaneous parametric down conversion (SPDC).

The experimental scheme adopted a nonlinear process, “quantum-injected optical parametric amplification”, which, by a linearized cloning process maps the quantum coherence of a single particle state, i.e., a microqubit, onto a macroqubit consisting of a large number M of photons in quantum superposition: Figure 7. Since the adopted scheme was found resilient to decoherence, a MQS demonstration was carried out experimentally at room temperature with $M=10^{4}$. The result led to an extended study of quantum cloning, quantum amplification, and quantum decoherence. Several experiments have been carried out, such as the test of the “nosignaling theorem”. In addition, the consideration of the microqubit-macroqubit entanglement regime has been extended to macroqubit-macroqubit conditions. The MQS interference patterns for large M are revealed in the experiment and bipartite microqubit-macroqubit entanglement was also demonstrated for a limited number of generated particles. For a complete description of this activity the reader can refer to [69].

## 8. Summary and Perspectives

The original work by Bennett et al. [1] has rapidly triggered a large number of investigations [10] for a broad range of applications [12]. Teleportation schemes were shown to enable new approaches for universal quantum computation [15,70], in particular as one-way quantum computers [71]. From the experimental perspective, numerous achievements have been reported on photonic platforms proving the feasibility of the scheme already with state-of-the-art technology. After the first demonstrations in 1997–1998 [16,17], one further proof appeared with the unconditional teleportation of optical coherent states with squeezed-state entanglement [26]. Later on, [72] provided a proof of the nonlocality of the process and of entanglement swapping. One year later, as shown in the previous sections, [18] teleported qubits were encoded in vacuum–one-photon states. The new century witnessed a worldwide race towards more complex implementations. In 2004, a single-mode discrete teleportation scheme using a quantum dot single-photon source has been demonstrated [73] based on the scheme of [18]. At the same time, several experiments addressed teleportation over larger distances [20,21,22,39]. Teleportation was also reported on squeezed entangled states: We refer to the review [12]. To bridge the gap between discrete and continuous variables, a hybrid approach has been recently reported [74]. Finally, achievements on photonic teleportation have been demonstrated with the first implementation on integrated circuits [75], as well as schemes with simultaneous teleportation of multiple degrees of freedom [27] and teleportation of qudits [76].

The past decade has seen a strong effort directed towards the development of matter-light interfaces as building blocks for quantum computation and communication, where entanglement between single-photon states and atomic ensembles represents an effective solution. The last few years have seen the implementation of quantum teleportation in scenarios of growing complexity. The following step is to exploit these results in order to achieve quantum networks over large distances thanks to the adoption of quantum repeaters. Concerning the research effort, the Quantum Information Lab is currently focused on experimental quantum causality. A promising direction is to exploit teleportation and entanglement swapping within such a framework [77].
